# Motor Imagery to Facilitate Sensorimotor Re-Learning (MOTIFS) after traumatic knee injury: study protocol for an adaptive randomized controlled trial

**DOI:** 10.1186/s13063-021-05713-8

**Published:** 2021-10-21

**Authors:** Niklas Cederström, Simon Granér, Gustav Nilsson, Rickard Dahan, Eva Ageberg

**Affiliations:** 1 Department of Health Sciences, Lund University, Margaretavägen 1B, 222 40 Lund, Sweden; 2 Department of Health Sciences, Lund University, PO Box 157, 221 00 Lund, Sweden; 3 Department of Psychology, Lund University, Allhelgona Kyrkogata 16a, 223 62 Lund, Sweden; 4 Malmö Idrottsklinik, Kalendegatan 20, 211 35 Malmö, Sweden; 5 Kulan Idrottsskadecentrum, Eric Perssons väg 5, 217 62 Malmö, Sweden

**Keywords:** Knee injury, Rehabilitation, Exercise therapy, Return to recreational activities, Psychology, Sports psychology

## Abstract

**Background:**

Treatment following traumatic knee injury includes neuromuscular training, with or without surgical reconstruction. The aim of rehabilitation is to restore muscle function and address psychological factors to allow a return to activity. Attention is often on rehabilitation of knee function, but deficiencies often persist. Specific interventions addressing psychological factors are sparing with varying degrees of success. We have developed a novel training program, MOTor Imagery to Facilitate Sensorimotor Re-Learning (MOTIFS), which integrates simultaneous psychological training into physical rehabilitation exercises. The MOTIFS model individualizes rehabilitation to increase central nervous system involvement by creating realistic and relevant mental images based on past experiences. We hypothesize that a 12-week MOTIFS training intervention will improve psychological readiness to return to activity and muscle function to a greater extent than current neuromuscular training (Care-as-Usual).

**Methods:**

This pragmatic 1:1 single assessor-blinded adaptive cumulative cluster-randomized controlled trial will include 106 knee-injured people with a goal of returning to physical activity. Participants are randomized to either the MOTIFS or Care-as-Usual condition. Primary outcomes are the ACL Return to Sport after Injury Scale and change in injured leg hop performance in a side hop task from baseline to 12 weeks. Secondary outcomes include patient-reported outcomes and assessment of muscle function using a hop test battery and Postural Orientation Errors at 12-week follow-up. At 12-month follow-up, patient-reported outcomes are assessed. A sub-group (7-10 in each group) will be interviewed to gain insight into experiences of rehabilitation.

**Discussion:**

Strengths of this trial include that it is a randomized and pragmatic trial examining commonly under-studied aspects of rehabilitation following a knee injury. The model uses the patient as a reference, creating simultaneous psychological and physical training exercises with easily adopted principles for clinical practice. Limitations include that blinding is limited due to study design, and shifting the clinical paradigm to a more holistic model is a challenge. If successful, the MOTIFS model has implications for a clinically useful, individualized, and patient-relevant method of improving rehabilitation outcomes by integrating psychological training into physical training.

**Trial registration:**

ClinicalTrials.gov NCT03473821. Registered March 22, 2018, with ethical approval that has been granted (Dnr 2016/413, Dnr 2018/927).

**Trial status:**

Trial Status: Protocol Version is 2020, Dec 10 – Version 1

**Supplementary Information:**

The online version contains supplementary material available at 10.1186/s13063-021-05713-8.

## Background

Treatment following a traumatic knee injury, such as to the anterior cruciate ligament (ACL), [1] meniscus [2], or patella [3], includes training with or without surgical intervention, with the goal of returning to pre-injury or modified activity levels. Despite current best-evidence treatment, many knee-injured people have poor patient-reported outcomes, such as perceived poor knee function and fear of re-injury, which are associated with poor rehabilitation outcomes in terms of lower return to physical activity [4]. Persistent sensorimotor deficiency has also been shown in the form of reduced muscle strength and hop performance [5] and undesirable postural orientation [6] in people with an ACL injury. In people with meniscal tears, reduced knee function and strength have been observed [7]. Poor muscle function in terms of limb asymmetry between the injured and uninjured legs in hop performance is associated with a lower probability of returning to pre-injury activity levels [8]. Reaching normal muscle function is, therefore, commonly considered an important milestone in returning to desired physical activity levels [9]. A meta-analysis and systematic review showed that people that returned to normal or nearly normal knee function (≤10% side-to-side difference) following ACL injury were more likely to return to activity, though only 65% of the total ACL-injured population returned to their preinjury level of sport, and 55% returned to competitive level sport [8]. Based on these results, factors other than muscle function are likely important for return to activity, and/or rehabilitation does not sufficiently prepare people for return to sport or physical activity.

Recent best-evidence recommendations have suggested that rehabilitation programs should focus on rehabilitation of the physical function of the knee and address psychological factors in order to improve the quality of life and prevent subsequent knee injuries/problems [9]. However, the majority of attention is on rehabilitation of physical function of the knee, using neuromuscular and strength training to reduce joint effusion, increase range of motion, and improve muscle strength, hop performance, and control of movements in weight-bearing positions [9]. Importantly, both physical and psychological factors are linked to rehabilitation outcomes. A systematic review and meta-analysis have shown that better muscle function such as hop performance, as well as positive psychological responses was associated with a higher likelihood of return to sports [8]. Research has also shown that higher self-efficacy, reduced fear of re-injury, and greater satisfaction with knee function are associated with better knee function and higher likelihood of return to physical activity [10]. Increasing focus on psychological interventions is important, as a knee injury is not purely physical, but requires a more holistic approach.

In uninjured people, psychological interventions both improve psychological outcomes [11] and increase central nervous system activation [12]. This increased activation could potentially address plastic changes in the brain, a suggested underlying cause of persisting sensorimotor deficiencies in people with an ACL injury [13]. Thus, a knee injury represents not only a musculoskeletal injury, but a neurophysiological dysfunction which may result from damage to the somatosensory system [14]. Specific psychological interventions to improve rehabilitation outcomes following knee injury are, however, sparing and with varying degrees of success, often performed completely separate from physical interventions. A systematic review of only four studies on psychosocial interventions in addition to separate knee rehabilitation, using static guided imagery and relaxation, or watching coping or therapeutic videos, noted improved self-efficacy, but with conflicting results for fear of re-injury/re-injury anxiety [15]. The mechanisms for these associations are unknown, which warrants more thorough investigation into other psychosocial interventions to optimize treatment outcomes. A potential solution may be more holistic rehabilitation programs in which consideration is given to the localized physical trauma, as well as to the central nervous system changes and the overall psychological aspects of the injury.

Currently, it is not known which psychological intervention has the greatest potential to improve outcomes in injured people. In uninjured people, sports psychologists use a set of training tools known as psychological skills training to enhance performance. The most common techniques used include goal-setting, arousal regulation, self-talk, and imagery [16]. Imagery is the practice of using one’s own memories and experiences to create a mental picture of a situation, without actually executing the movement [17]. Imaging while focusing on contextual, tactical, and kinesthetic aspects is referred to as “motor imagery” [17]. This can be done while sitting or standing still with little to no physical movement, known as static imagery. Dynamic motor imagery (DMI) is the practice of imaging a specific situation while simultaneously physically mimicking part or all of the movement in order to increase realism [17]. The aim is to improve performance by creating a functionally equivalent image of a relevant and activity-specific movement [18]. Functional equivalence in this sense means that the movement being performed is as close as possible to actual execution by focusing on Physical, Environmental, Task, Timing, Learning, Emotion, and Perspective (known by the acronym PETTLEP) [18].

In uninjured athletes, physical training with no imagery, only static imagery, and only DMI have all resulted in performance enhancement, although performance benefits appear to be optimized when DMI is combined with physical training [19]. According to Self-Determination Theory, motivation can be increased using an autonomous contextual decision-making process, individual connection to the activity, and by applying one’s own competencies [20], all of which can be accomplished using DMI.

Shifting the focus to individual relevance may influence motor learning and performance of experienced-based movements, according to the OPTIMAL theory of motor learning [21]. This is also supported by the theory of re-investment, which states that motor execution can be disrupted through attempts at conscious control [22]. For example, focusing on how to execute a situation-based (i.e., soccer) task, rather than on movement technique, relies more on automatic processes to create efficient movement [22]. A more external focus also creates an understanding of the applicability of the movement, shifting motivation from extrinsic (outcome-oriented) to intrinsic (inherent satisfaction), which is important for adherence [23]. Adherence may then also influence physical outcomes by virtue of having performed the exercises [24].

Integrating DMI into knee-injury rehabilitation constitutes a potential alternative to improve the effectiveness of rehabilitation by targeting a gap in the rehabilitation literature. This allows for specific interventions that can be used in a clinical environment which aim to address and affect physical, neurological, and psychological aspects.

This study protocol describes an ongoing adaptive randomized controlled trial, the aim of which is to compare the efficacy of rehabilitation with integrated dynamic motor imagery, and rehabilitation alone (care-as-usual) in terms of improving both muscle function and patient-reported outcomes in physically active males and females with a traumatic knee injury and with a goal of returning to physical activity. This approach of integrating physical and psychological aspects in rehabilitation takes a holistic perspective of the injury, in contrast to an isolated physical perspective in care-as-usual rehabilitation.

The main hypothesis is that 12 weeks of rehabilitation with integrated dynamic motor imagery will improve patient-reported psychological readiness to return to physical activity and objective muscle function to a greater extent than CaU rehabilitation.

Secondary aims include examining the effect of rehabilitation with integrated DMI on patient-reported outcomes, performance in a hop battery, and postural orientation. The exploratory aims include a phenomenological interview with patients to gain further insight into the perceived experiences throughout the rehabilitation process.

## Methods

### Study design

**Fig. 1:**
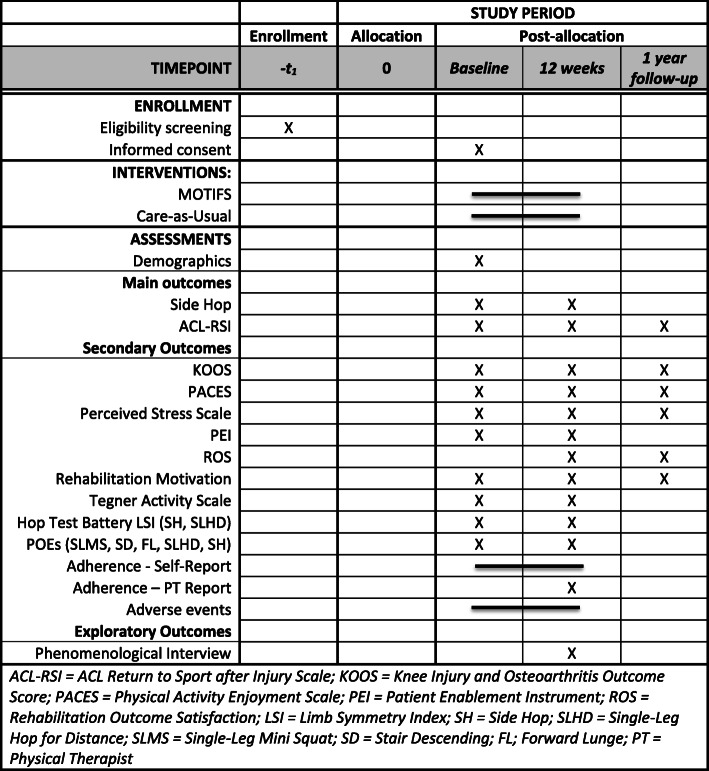
Protocol schedule of forms and procedures

The design of this study is a 1:1 single assessor-blinded cumulative adaptive cluster-randomized controlled trial, conforming to the Consolidated Standards of Reporting Trials (CONSORT) statement extension for pragmatic trials [25], with the intervention administered in real-world settings and conditions (trial pragmatism illustrated in Additional File 1). The cumulative adaptive design allows for modification upon evaluation of a subgroup in order to more accurately determine the required sample size, as well as whether changes need to be made to the method before the continuation of the trial [26]. Items from the World Health Organization Trial Registration Data Set are available on the ClinicalTrials.gov registration page (NCT03473821). This protocol adheres to the Standard Protocol Items: Recommendations for Interventional Trials (SPIRIT) guidelines [27], procedures of which are outlined in Fig. 1 (SPIRIT checklist available as Additional File 2). Ethical approval has been granted by the Regional Ethical Review Board in Lund, Sweden (Dnr 2016/413, Dnr 2018/927).

### Participants and randomization

Approximately 106 participants are recruited according to inclusion criteria (Table 1) from physical therapy clinics in Sweden. Physical therapists (PTs) fulfilling inclusion criteria (Table 1) from these clinics are included in the trial. The eligibility criteria for both PTs and participants allow for the recruitment of a sample of knee-injured people and their PTs representative of the population. To avoid contamination, participating clinics are block randomized by a third party using a random numbers generator and sealed opaque envelopes to either the experimental or CaU condition.

**Table 1:** Participant and physical therapist inclusion and exclusion criteria

Participants	
Inclusion	Exclusion
- Men and women 16 years or older - Traumatic knee injury (e.g., ligament, meniscal, patellar, or chondral injury), with or without surgical intervention, and involving one or more knee structures - Currently undergoing physical therapist-supervised rehabilitation training - Have reached the stage in their rehabilitation which includes single-leg hop training, as well as a change of direction - Active before the injury in recreational or competitive physical activity, with a goal of returning to physical activity	- Any degenerative knee disorder (e.g., osteoarthritis), or other disease or disorder (e.g., spine disorder, neurological disease) overriding the traumatic knee injury - Reached end-stage rehabilitation (i.e., have undergone a return to physical activity evaluation by their physical therapist, or is estimated to return less than 12 weeks from potential inclusion date) - Do not understand the languages of interest (Scandinavian languages, English)
**Physical therapist**	
**Inclusion**	
- At least 2 years’ experience of treatment with training for musculoskeletal disorders	
- Treat patients with a knee injury on a weekly basis	

Clinics, as opposed to participants, are randomized in order to avoid familiarization with both conditions within the same clinic or the individual PT, a potential source of contamination which could influence intervention adherence and therefore skew results. In accordance with the Swedish health care system, participants select their clinic, and clinics do not actively advertise in order to recruit patients (making randomization at the participant level impossible), so self-selection of a treatment arm is theoretically random and blinded. PTs are given information on the methodology of their assigned intervention arm to allow them to recruit participants from their population.

Due to the nature of the design, as well as practical constraints (i.e., necessity of familiarity with intervention, staff availability), neither the study coordinator (SC), participants, nor PTs can be blinded. As a result, the test leader is blinded to the treatment condition and un-blinding is not permissible. Final data analysis is performed by a blinded third party in order to avoid potential bias.

### Procedure

All knee injuries from the PTs patient pool are reported to the SC during weekly contact, in which the SC reminds PTs of inclusion criteria and actively seeks information on knee-injured participants in order to approach all potential participants in the patient pool. The number of patients approached and invited (i.e., eligible, ineligible, and declined participation) will be recorded. The SC contacts potential participants that have consented to a pre-screening interview, during which information is provided, inclusion, and exclusion criteria are verified, and, if eligible, the participant is invited to take part in the study. Information provided to potential participants includes only that there is a “mental training intervention” with no details which could be used to replicate the intervention, adhering to Swedish ethical guidelines for participant informed consent. Following pre-screening, the SC has regular contact with participants and physical therapists in order to receive updates on patient progression through rehabilitation. This ensures proper inclusion timing and reduces the risk of late inclusion by reducing dependency on physical therapists with regard to inclusion criteria, as well as ensuring that eligible participants are included in the trial. Rehabilitation during this period is provided according to rehabilitation protocols prescribed at the discretion of the PT until the participant is able to complete single-leg hop tasks, ensuring proper inclusion timing. When the hop ability criterion is confirmed by the PT, written information is provided, and baseline testing takes place, during which the test leader collects informed consent (Additional File 3 – Informed Consent Materials). The intervention then begins, implementing either CaU or MOTIFS training in the clinical setting during treatment. The intervention period is 12 weeks, as studies have shown that physical [28] and psychological [29] factors can be improved in this time span. MOTIFS training accounts for approximately 20 min [30] in a typical 60-min training session. At least 3 supervised or at-home training sessions are performed per week, whereof at least 6 are supervised by a participating PT during the intervention period, resulting in an intervention dose of 36 possible training sessions, with intensity prescribed by the PT. Participants and PTs are made aware of the possibility to end their involvement in the study. Participants may remove themselves at their discretion, or worsening symptoms may require the PT to change treatment strategy. To ensure that participants complete self-report questionnaires, telephone contact may be initiated.

#### Care-as-usual intervention

The control condition includes 12 weeks of “care-as-usual” training (CaU), in which participants receive treatment at the discretion of the participating PT according to commonly used neuromuscular training practices in physical therapy settings in southern Sweden. These typically aim to improve motor control and achieve dynamic stability in people with a knee injury [9]. Emphasis is on the quality and efficiency of movement, with little to no structured psychological training (Additional File 4: Appendix 1.3 [Appendix Table 1 – Care-as-Usual column]).

Participants in the CaU condition are contacted by the SC by telephone three times during the intervention period to ensure adherence to training and provide rehabilitation updates. This is done in order to maintain equal contact as compared with the MOTIFS condition.

##### Motor Imagery to Facilitate Sensorimotor Re-Learning (MOTIFS)

We have developed Motor Imagery to Facilitate Sensorimotor re-learning (MOTIFS), a novel training model designed to integrate psychological training into care-as-usual rehabilitation exercises.

##### Physical background to MOTIFS

The MOTIFS model integrates DMI into care-as-usual neuromuscular training principles. The majority of training is comprised of complex movements with greater speed and intensity in multiple planes at the discretion of the participating PT, for example, hop, change of direction, and pivoting exercises.

##### Psychological background to MOTIFS

An external focus of attention, focusing on something outside the body, may be more beneficial for the development of motor control and movement efficiency than an internal focus on one’s own body and movements [21]. Instructing a person to reach their knees towards a point on the wall during a squat creates an external focus, as opposed to maintaining a knee-over foot position, which is internal. The external focus on specific and experience-based skill practice facilitates automatic processes [21], allowing a PT to help a patient to more easily and correctly execute familiar and relevant movements, such as reaching for a ball in a defensive situation, as opposed to merely reaching towards the wall. Therefore, rehabilitation with integrated functionally equivalent simulations of activity-specific situations may aid in making proper movement patterns more automatic. This, along with implications for motivation [22] and enjoyment and feelings of control [31], indicates that the holistic MOTIFS model may improve psychological and physical rehabilitation outcomes.

##### MOTIFS model principles

**Fig. 2:**
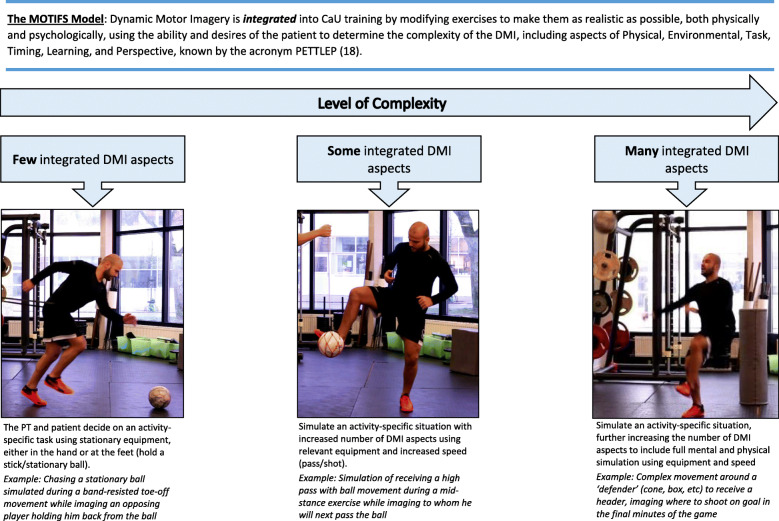
Modulating complexity of the MOTIFS DMI to adapt to the needs and desires of the patient in question

MOTIFS training uses principles based on the PETTLEP model of DMI to individualize psychological training based on a person’s own experiences (see Appendix 1.4 for details) [18]. The MOTIFS principles (Table 2; Appendix 1.2) provide clinical guidelines to determine how to physically and psychologically individualize treatment. Following discussions with a patient, the level of complexity of the integrated DMI can be modified (Fig. 2) in order to achieve external and context-specific psychological goals, as well as physical and rehabilitation goals. Shared decision-making is employed to ensure activity-relevant applicability of the PT-prescribed rehabilitation movement in order to increase patient involvement in the design of the exercise itself. This process is repeated throughout rehabilitation to integrate MOTIFS training into CaU exercises, adapting to fit the physical and psychological needs of the individual patient.

**Table 2:** Principles utilized in the MOTIFS model

The MOTIFS model modifies Care-as-Usual rehabilitation to create sport-specific exercises individualized to the needs and goals of the patient using as many aspects of PETTLEP motor imagery as possible		
**1**	**Discuss**	A realistic scenario is created through discussion between the PT and patient based on the rehabilitation movement and meaningful and relevant aspects of the participant’s prior experiences
**2**	**Create**	An exercise is created by both the PT and patient which simulates the individualized and realistic scenario
**3**	**Execute**	The activity-specific rehabilitation movement with integrated dynamic motor imagery is executed as realistically as possible, including physical and mental simulation, equipment, and a full-body and context-specific follow-through
**4**	**Evaluate**	Upon completion, the patient and PT discuss and evaluate the simulation and repeat the process, with modifications to increase realism and meaning, including a progression towards physically and psychologically realistic and sport-specific training exercises.

The MOTIFS model is a clinically feasible training model with implications for both physical and psychological rehabilitation. The aim is to improve knee strength and postural control and re-learn activity-relevant movements that are simultaneously more meaningful and enjoyable for the patient.

##### MOTIFS intervention

Care-as-usual rehabilitation exercises with integrated DMI constitute the MOTIFS model, the premise of which is that aspects of DMI are integrated into commonly used knee rehabilitation movements, as prescribed by the treating PT. The research team (NC, SG, GN, RD, EA) lead a workshop in which a PT specializing in neuromuscular training (GN) and a sport psychologist specializing in DMI (NC) train the physical therapists to deliver the MOTIFS training model. The workshop consists of a 5-h session in which physical therapists in the experimental group receive theoretical and practical instruction in the proper methodology of the MOTIFS model. Detailed information, including a film and a manual, is provided to aid in the effective implementation of the intervention in the clinical setting. It is also ensured that relevant equipment is available to patients (i.e. balls or sticks). Additional support workshops are held throughout the intervention period.

Participating PTs that have been educated on the implementation of the MOTIFS model explain, individualize, and administer the intervention to participants during face-to-face rehabilitation training sessions either individually or in small groups. This is supplemented by information and three face-to-face support visits by the SC with the patient, together with the PT, in order to allow for discussion and questions regarding the proper utilization of the MOTIFS model during supervised and at-home training sessions. These visits ensure proper patient fidelity and also assess whether the physical therapists are correctly using the MOTIFS model. Participants are instructed to train using the principles of the MOTIFS model, in which PT-prescribed physical exercises with simultaneous DMI are performed based on the participant’s desired activity-specific situation. MOTIFS training is applied in relevant situations and allows for separation between activity-specific functional movements and other aspects of rehabilitation that the PT deems necessary such as warm-up, strength training, balance training, mobility exercises, and/or cool-down.

#### Outcome assessment

Physical and patient-reported outcomes are assessed at baseline and 12 weeks; patient-reported outcomes are assessed 12 months post-inclusion (Fig. 1). Prior to the testing occasion, self-report questionnaires are distributed using the REDCap electronic data capture tool [32], hosted by Lund University. Demographic data (age, gender, height, weight, treatment type, and injury history) and physical testing data (Table 3) are collected by an experienced test-leader either at the participants’ respective clinics or at the university lab according to a data collection form including testing procedures (further information is available in the data management plan, available at the ClinicalTrials.gov registration page: NCT03473821). Outcomes are assessed at 12 weeks post-inclusion to assess physical and psychological readiness when a PT would likely consider a return to activity testing, and at 12 months to assess long-term rehabilitation outcomes.

**Table 3:** Outcomes assessment—description of types of outcome and the data used for analysis

Outcome		Type	Outcome data
Patient-reported outcomes	ACL-RSI^a^	12 item self-report scale (0–100 for each item)	0% (worst) to 100% (best) mean of summed total score
	KOOS	The normalized score for subscales: pain, symptoms, sports and recreational activity, activities of daily life, and quality of life	0 (worst) to 100 (best)
	PACES	18-item self-report scale (11 items reverse-scored)	18 (worst)–126 (best)
	PSS	10-item self-report scale	0 (best) to 40 (worst); 0–13 = low, 14–26 = moderate, 27–40 = high stress
	PEI	6-item self-report	0 (worst) to 12 (best)
	ROS	1-item self-report scale	Satisfied (3 or 2), mostly satisfied (1), or dissatisfied (0, −1, −2, or −3)
	RM	3-item self-report scale	0 (worst) to 10 (best); raw data presented
	TAS	3-item self-report scale (pre-injury, current, future)	1 (low activity)–10 (very high activity)
Physical testing	SH^a^	Number of completed hops	Change in the injured leg
	Hop test battery	SLHD-3 repetitions or until improvement <10cm; distance hopped (cm)	Change in the injured leg, change in the uninjured leg, LSI
		SH-number completed hops in 30 s	
	POEs	SLMS-5 repetitions; POEs assessed throughout the entire movement	Total POEs score
		SD-5 repetitions; POEs assessed on loading leg throughout entire movement until both feet are on the floor	
		FL-3 repetitions; POEs assessed on the front leg from landing until maximum flexion of the knee	
		SLHD-POEs assessed from landing until the knee is extended	
		SH-7 repetitions; POEs assessed during landing in 3 medial and 3 lateral jumps	
Adherence		Self-report questionnaire	Number and duration of training sessions
		Physical therapist report	Number of sessions attended
Adverse events		Self-report questionnaire	Number and type of adverse events, if any
Exploratory outcomes		Phenomenological interview	Coded interview data

*ACL-RSI* ACL Return to Sport after Injury Scale, *KOOS* Knee Injury and Osteoarthritis Outcome Score, *PACES* Physical Activity Enjoyment Scale, *PSS* Perceived Stress Scale, *PEI* patient enablement instrument, *ROS* rehabilitation outcome satisfaction, *RM* rehabilitation motivation, *TAS* Tegner Activity Scale, *SH* side hop, *SLHD* single-leg hop for distance, *LSI* Limb Symmetry Index, *POEs* postural orientation errors, *SLMS* single-leg mini squat, *SD* stair descending, *FL* forward lunge, *ADL* activities of daily life

^a^Main outcome

#### Main outcomes

This trial includes two main outcomes. Psychological readiness to return to sport is evaluated using the Swedish version of the ACL-Return to Sport after Injury scale (ACL-RSI), which includes an emotional response, confidence, and risk-appraisal when returning to sport after an ACL injury [33, 34]. Physical function is evaluated using the change in number of hops in the injured leg from baseline to 12 weeks for the side hop task.

#### Secondary outcomes

##### Patient-reported outcomes

ACL-RSI scores are summed for a scale from 0 to 100%, with higher scores representing more positive psychological reactions [33]. The Swedish version of the Knee Injury and Osteoarthritis Outcome Score (KOOS) is used to evaluate self-reported knee function [35, 36]. Enjoyment is measured using the Physical Activity Enjoyment Scale (PACES) [37], using an unpublished version translated to Swedish by the authors. Stress is measured using the Swedish version of the Perceived Stress Scale [38, 39]. The patient’s ability to understand and cope with a disease or injury is assessed using the Patient Enablement Instrument [40, 41]. Rehabilitation outcome satisfaction is measured using one unvalidated question (“if you were to spend the rest of your life with your knee function just the way it has been in the last week, would you feel…”) [42]. Rehabilitation motivation is evaluated using three questions adapted from a previous study: “how important is it that you return to your previous activity level?”, “do you think it is possible for you to return to your previous activity level?”, and “how much time and effort are you willing to invest to return to your previous activity level?” [4]. The Tegner Activity Scale [43] is used to assess preinjury, current, and desired future activity level. All questionnaires are available in English.

##### Physical testing

Physical testing is performed in order of difficulty, with activities resembling daily life (single-leg mini squat) being performed first, progressing to more demanding functional tasks (side hop). Tasks are performed with shoes unless otherwise stated. The tasks are described below according to the test batteries in which they will be analyzed.

##### Hop test battery

The hop test battery [6], consisting of two functional tasks (single-leg hop for distance and side hop) performed on both legs, is used to evaluate muscle function. The single-leg hop for distance is done by standing on one leg and performing a maximal jump forward, landing with control on the same leg, and maintaining control for 2–3 s. Failure to maintain landing position results in a disqualified hop and the hop will be repeated. The side hop task is performed by jumping on one leg from side to side over two parallel lines taped 40 cm apart on the floor as many times as possible in 30 s [44]. The number of successful hops without touching the line is recorded. Results are expressed as change in the injured leg, change in the uninjured leg, and limb symmetry index (LSI; LSI = (injured leg/uninjured leg) x100) individually and summed.

##### Postural orientation errors

Postural orientation errors (POEs) are assessed using a test battery consisting of five functional tasks (single-leg mini squat, stair descending, forward lunge, single-leg hop for distance, and side hop) that range from resembling daily life to more demanding activities [45]. The single-leg mini squat [46] is performed without shoes or fingertip support by lifting one leg and lowering the body until the active knee reaches approximately 60° of flexion, then the individual returns to full extension. The stair descending [47] task is performed without shoes by standing on a 30-cm box or raised platform and stepping down from the platform one foot at a time, as though walking down a set of stairs. The forward lunge is performed by standing on a starting line, taking a step forward, and flexing the knee to approximately 90°, then extending to return to the starting position [6]. The single-leg hop for distance and side hop is performed as described above. Tasks are video-recorded from a frontal view for later assessment of the participant’s postural orientation, presented as a total POE score [45].

##### Adherence

Adherence is measured using a weekly self-report survey regarding the number of completed supervised and at-home rehabilitation sessions, the approximate duration of at-home training sessions, and the approximate duration of MOTIFS training during at-home training sessions (final question answered only by the MOTIFS group). Physical therapist attendance records may be requested to confirm the number of completed supervised rehabilitation sessions over the intervention period. Adherence will be compared between groups to determine whether one treatment has higher adherence rates.

One unvalidated self-report question will be posed to participants in the MOTIFS condition in the adherence survey to assess whether they engaged in imagery during the execution of their rehabilitation exercises. The question was adapted from the Vividness of Movement Imagery Questionnaire [48]: “While doing my rehabilitation exercises, the sport-/physical activity-specific image that I create is:” with responses on a 5-point Likert scale ranging from 1 (“as vivid as though I were actually doing it”) to 5 (“not at all vivid”), with an additional option of not applicable if they did not perform imagery during their exercises. This adherence question also provides an indication of whether the physical therapists were prescribing MOTIFS training during physical rehabilitation sessions.

#### Exploratory outcomes

Upon completion of the intervention, a subgroup of approximately 10 participants in each group (or until saturation is reached) will undergo a phenomenological interview about their rehabilitation experience. Phenomenological interviews will also be conducted with physical therapists (approximately *n*=7–10 from each group) to provide insight into potential modifications to be made to the MOTIFS model. These will also provide information regarding how PTs in both the MOTIFS and CaU groups experience planning and executing rehabilitation to treat traumatic knee injury using their assigned intervention. The interviews, consisting of one question regarding their rehabilitation supplemented by probing follow-up questions, will be audio-recorded, transcribed verbatim, and coded according to phenomenological methodology. Results will be presented in future articles; this protocol will therefore not cover this in-depth.

### Statistical analyses

#### Sample size

Based on previous studies examining ACL-RSI [49] and side-hop function [50], a preliminary sample size was estimated, to be used in the first phase of this adaptive RCT, which resulted in *n*=106 participants. This included adjustments for potential cluster effects using a preliminary Intra-Cluster Correlation Coefficient (ICC). An approximation is required as comparable studies are unavailable to reliably perform a sample size estimation. This is necessary in this phase in order to evaluate whether an intervention effect is detectable and within practical limitations, which will thereafter inform more accurate sample-size estimations.

A new sample size will be calculated by a blinded third-party member of the data management committee using collected data from an interim analysis, therefore allowing for more accurate between-group differences in the main outcomes. Observed within- and between-cluster variance will also be calculated in order to more accurately estimate an Intra-Cluster Correlation Coefficient (ICC, denoted as *ρ*). This will be performed using the formula $\rho =\frac{s_b^2}{s_b^2+{s}_w^2}$ [51] in order to adjust for the fact that different clinics are included in the trial (i.e., potential clustering effects). The adjusted sample size will therefore be calculated using the formula $\frac{n_ik\left(1-\rho \right)}{k-{n}_i\rho }$ [52], along with an estimated 20% drop-out rate, in order to reach a power of 0.80, with significance set at *p*<0.025. As an estimated sample size has been calculated, the new sample size will only be adjusted up; that is, the number of participants included will not be less than *n*=106 required in the original sample-size estimation.

#### Adaptive design modification criteria

The mean scores of the main outcomes (side hop and ACL-RSI) will be compared in exploratory interim analyses once *n*=25 participants have been included in both groups. Analyses will be performed by a blinded third-party data management committee (DMC) and blinded to treatment arms until after an initial data analysis and interpretation. The purpose of the interim analysis is primarily to gain data with which a more reliable sample size can be calculated based on observed differences, as well as to identify potential benefits and harms which may thereby determine whether modifications are required. Therefore, differences in the main outcomes from baseline to 12 weeks will be compared using estimations from the previously defined clinically relevant differences as modification criteria, as both groups are expected to improve. Continuation without modification will be allowed if the between-groups difference in ACL-RSI is ≥5 points, and/or muscle function (LSI) in the side-hop test is ≥8 in favor of the MOTIFS group, each analyzed separately. These estimates correspond to a small to medium effect size (Cohen’s *d*≈0.1–0.3) based on previously defined clinically relevant values and will be used to determine the potential for the MOTIFS model to exhibit an effect. However, as previous studies which provide enough data for reliable calculation are lacking, these effect sizes are theoretical estimations which require further data to confirm. The estimates take into account the small sample size in the interim analysis and serve as a guide to whether the differences exhibit a trend indicating acceptable power upon completion of data collection, without unnecessarily prematurely ending the trial. If these initial criteria are not met, difference in enjoyment (PACES) scores of approximately 5 points will be deemed acceptable, estimated based on studies examining the effect of enjoyment on physical activity adherence in uninjured people [53]. Increased adherence to rehabilitation training will likely lead to improved rehabilitation outcomes based on a dose-response relationship, so increased enjoyment is considered an acceptable criterion for the continuation of the trial. Upon completing the interim analysis, the DMC will determine whether these criteria are met, and/or whether modifications are necessary, and a new sample size calculation will be performed.

Modification recommendations will be made based on observed data to identify potential harms or an unrealistic new estimated sample size estimation (i.e., more than 100 patients in each group), for example. In this case, the method will be refined to ensure that the model’s efficacy is maximized according to the proposed outcomes (i.e., psychological readiness to return to sport and muscle function results) and that implementation is clinically relevant and feasible for the end-users. Pilot testing will then be performed again, examining a new pilot population a maximum of one additional time to evaluate whether a satisfactory level of clinically relevant power is achieved (i.e., continuation criteria are met), thereby justifying the continuation of the trial. Modifications will be informed by preliminary results from interim analyses of this cumulative adaptive trial examining both participant data and PT interview responses in the research team, DMC, and potentially discussions with a focus group comprised of end-users (PTs and patients). Following the initial interim analysis, the *p* value will be adjusted using a Bonferroni correction to take into account the risk of type-I error inherent in repeating the pilot phase.

#### Data analysis

The analysis will be done using the IBM SPSS statistical software package (IBM Corp., Armonk, New York, USA). Primary analyses of the entire sample will be done according to intention-to-treat principles. A dose-response analysis will be performed to determine effective adherence. Per-protocol analyses will be performed for those participants with at least 70% adherence (corresponding to two trainings per week), as a preliminary estimate of the effective number of training occasions. This is in order to eliminate cross-over and drop-out effects in the total sample, as well as to evaluate the training for those that have reached a pre-defined level of adherence deemed acceptable to have an effect. Sensitivity analyses will be performed to determine whether within-cluster correlations indicate potential effects due to being clustered, for example, by the clinic or by PT. Patient cross-over (i.e., patients changing clinics) is a low-risk possibility and will be recorded in case of occurrence. All outcome variables will be examined for normality using appropriate testing (e.g., Kolmogorov-Smirnov). Complete-case analyses are the primary analyses, and sensitivity analyses will be done depending on the number of missing values. Missing values are not expected to be extensive, and if less than 5% of data is missing, the whole data set will be analyzed along with a sensitivity analysis with appropriate imputed values. If between 5 and 40% of data is missing, appropriate multiple imputation methods will be used, which will be informed by blindly assessing results of the interim analysis, and a complete-case analysis will be done with appropriate sensitivity analyses. If over 40% is missing, a complete case analysis will be done with no sensitivity analysis.

Results will be analyzed using independent samples *t* tests to examine between-group differences of the change from baseline to a 12-week follow-up. Where appropriate, equivalent non-parametric Wilcoxon signed-rank testing will be used to compare between-group differences. The main analysis will focus on the two main outcomes, with secondary outcomes acting as support for these. Secondary outcomes therefore carry less weight and issues with potential error due to the number of evaluations will be discussed when results are presented.

Due to the low range and number of items of the perceived stress scale, Patient Enablement Instrument, and Tegner Activity Scale, as well as the fact that both groups are expected to improve, chi-squared tests will be used to compare within-subjects between-groups means. Rehabilitation motivation and outcome satisfaction will be presented as raw data due to the low number of items. Attrition analyses will also be performed.

### Adverse events/harms

Adverse events are recorded using weekly self-report questionnaires consisting of five questions, distributed using REDCap software. Three questions are directed towards training-related adverse events: “has your knee given way (that is, in the same way as when you injured your knee) in connection with rehabilitation training with your physical therapist or on your own?”; “has your knee swelled up in connection with doing your rehabilitation training with your physical therapist or on your own?”; “have you experienced more pain in your knee in connection with your rehabilitation training with your physical therapist or on your own?” Two questions are related to adverse events in general (i.e., non-training related or overall): “have you had a new knee injury (that made it so you had to use crutches or undergo another surgery in your knee)?”; “have you had any other sickness (influenza, cold, etc.) or injury (sprained ankle, back pain) that resulted in not being able to do your rehabilitation training with your physical therapist or on your own?” Giving way episodes and new injuries which require further medical care are considered serious.

### Data management

Upon collection, data is checked by the SC and entered into a data file stored on a secure server. In cases of missing data, non-response, or questionable data, the SC contacts the participant to clarify. Data monitoring is done by NC on a regular basis, and any abnormalities are discussed with the research team (NC, SG, EA). Final data analysis will be informed by a statistician and performed by an unbiased third-party statistician.

## Discussion

The research team is composed of experts in physical therapy and sports medicine (EA), sport psychology (NC, SG), and clinically active physical therapists with a master’s degree and 10 (GN) and 26 (RD) years of clinical experience. This experience and expertise, along with consultations with other clinically active physical therapists, informed the research questions and training model. The model was tested on a range of physically active people with knee injury undergoing physical therapy rehabilitation and subsequently modified where appropriate. Further testing was done with patients until the model was deemed acceptable by both the research team and end-users (physical therapists, patients). Participating PTs in the study were also consulted in regards to recruiting for and executing the intervention, and changes were made to accommodate this. Interviews will be conducted with both PTs and patients to further explore the clinical relevance and applicability of the MOTIFS model.

The strengths of this trial include that it is randomized and pragmatic, exploring the MOTIFS model implemented under clinically relevant conditions experienced by end-users (i.e., both PTs and patients), as opposed to in a lab under ideal conditions. The MOTIFS model utilizes shared decision-making between the PT and the patient in order to create a unique care protocol based on the needs and desires of the individual that can be modified to fit any clinical rehabilitation setting. For this first RCT in which the MOTIFS model will be evaluated, patients in the later stage of rehabilitation are included. Principles of the MOTIFS model are applicable to patients in all phases of rehabilitation and in the treatment of other musculoskeletal injuries which may be the focus of future trials.

Limitations include that only the test leader is blinded to condition assignment due to the nature of the design. As the study coordinator is not blinded, bias cannot be ruled out during recruitment. Information to participants includes a brief description of the aims of the study in line with Swedish ethical guidelines, which may influence participants’ decision to participate or their behavior during the trial (i.e., if aware of a mental training intervention, more focus may be placed on mental aspects). However, as the participants receive no specific information, and imagery is assessed as a compliance measure, this is not seen as problematic. A further limitation includes that previous studies do not provide relevant data for sample size calculation. Mean differences, standard deviations, and variance are therefore theoretical, leading the authors to use an adaptive design in order to collect data which may be used for sample-size re-calculation. Simulation analyses, which may provide more accurate estimations, were not used to estimate sample size as these theoretical and estimated values would not provide reliable results. This highlights the adaptive nature of this trial and having collected data will provide information necessary to perform reliable sample size and power calculations for future research. This trial presents challenges in that it introduces the more holistic MOTIFS model, which shifts the clinical paradigm and places more focus on the individual and their context-specific needs from a psychological perspective. In order to aid in this, individual visits with the SC, as well as guidance and workshops, will smooth the transition to the novel MOTIFS training model.

Along with publication in a peer-reviewed journal, results of the study will be communicated via a popular science article in order to inform potential end-users of the outcome of this trial, with the aim of making the MOTIFS model readily available for use by end-users.

### Trial status

This study was registered on ClinicalTrials.gov (NCT03473821) in March 2018. Enrollment of physical therapy clinics began in October 2017. Enrollment of participants began in March 2018, and is estimated to be completed in December 2021. At the time of writing, 35 participants have been included, and 11 have preliminarily agreed to participate. Data collection is estimated to be completed in spring 2022.

## Supplementary Information

**Additional File 1.** PRagmatic Explanatory Continuum Indicator Summary (PRECIS-2) Figure evaluating trial pragmatism

**Additional File 2.** Spirit Checklist - Completed checklist identifying fulfillment of SPIRIT guideline requirements

**Additional File 3.**Informed Consent Materials; Informed consent sheet supplied to participants (translated from Swedish to English by the first author; original Swedish version available upon request)

**Additional File 4:** Appendix 1 - Detailed description of Care-as-Usual and PETTLEP training, and practical use of the MOTIFS model

## Data Availability

The datasets generated for the present study are not currently publicly available, as data collection is ongoing, but may be available from the corresponding author on reasonable request, provided that analyses have been completed. A data management protocol is available on ClinicalTrials.gov.

## Abbreviations

ACL: Anterior cruciate ligament
DMI: Dynamic motor imagery
CaU: Care-as-usual
CONSORT: Consolidated Standards of Reporting Trials
SPIRIT: Standard Protocol Items: Recommendations for Interventional Trials
PT: Physical therapist
SC: Study coordinator
MOTIFS (MOTIFS): Motor Imagery to Facilitate Sensorimotor re-learning
PETTLEP: Physical, Environmental, Task, Timing, Learning, Emotion, Perspective
ACL-RSI: Anterior Cruciate Ligament Return to Sport after Injury scale
KOOS: Knee Injury and Osteoarthritis Outcome Score
PACES: Physical Activity Enjoyment Scale
POEs: Postural Orientation Errors
