# Supplementary material for: Motor Imagery to Facilitate Sensorimotor Re-Learning (MOTIFS) after traumatic knee injury: study protocol for an adaptive randomized controlled trial
Source: Trials. 2021 Oct 21;22:729. doi: 10.1186/s13063-021-05713-8 (PMC8532360; doi:10.1186/s13063-021-05713-8)
Supplement: Supplementary file 1 — Additional File 1. PRagmatic Explanatory Continuum Indicator Summary (PRECIS-2) Figure evaluating trial pragmatism [file 13063_2021_5713_MOESM1_ESM.pdf]

Additional File 1 – PRECIS-2 Figure evaluating pragmatism
Cederström et al - MOTIFS Study Protocol

The PRagmatic Explanatory Continuum Indicator Summary (PRECIS-2) tool evaluates pragmatism in a trial, thereby informing study design in terms of real-world applicability.(1) The figure visually represents the level of pragmatism in a trial on a scale based on author-reported scores in 9 domains on a scale from 1 (explanatory) – 5 (pragmatic). Rationale for each domain score is available on the PRECIS-2 website (https://www.precis-2.org/Trials/Details/528).

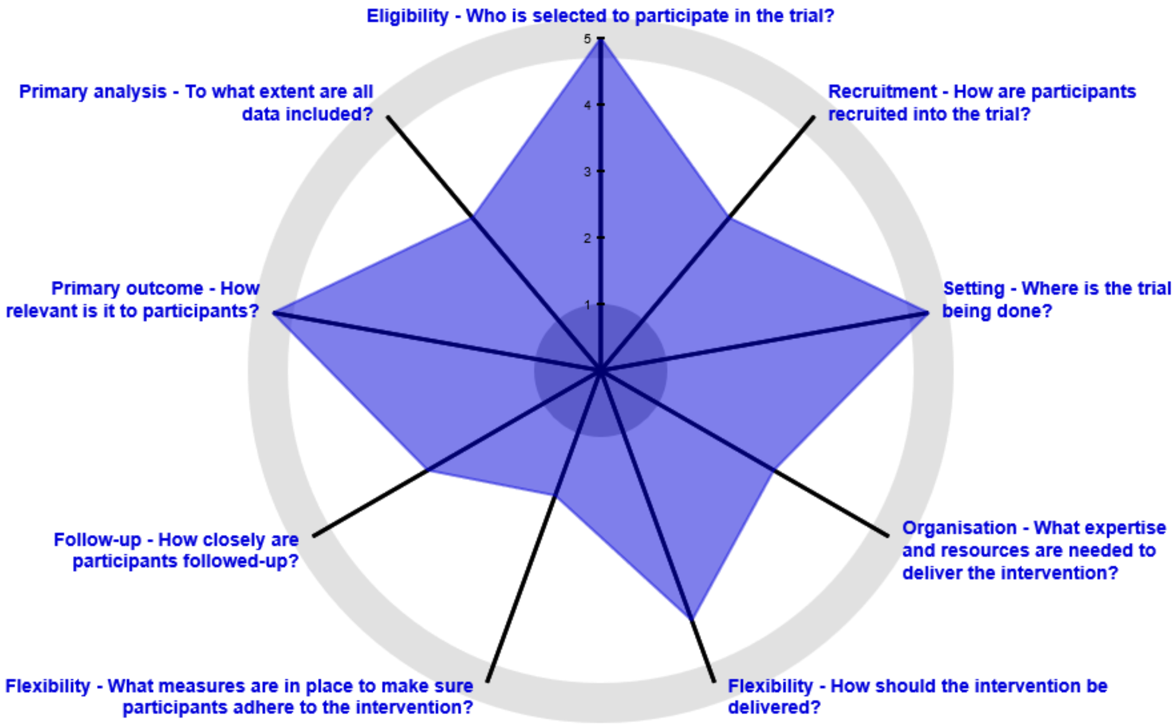

1. Loudon K, Treweek S, Sullivan F, Donnan P, Thorpe KE, Zwarenstein M. The PRECIS-2 tool: designing trials that are fit for purpose. Br Med J. 2015;350:h2147.
