# Supplementary material for: Motor Imagery to Facilitate Sensorimotor Re-Learning (MOTIFS) after traumatic knee injury: study protocol for an adaptive randomized controlled trial
Source: Trials. 2021 Oct 21;22:729. doi: 10.1186/s13063-021-05713-8 (PMC8532360; doi:10.1186/s13063-021-05713-8)
Supplement: Supplementary file 3 — Additional File 3.Informed Consent Materials; Informed consent sheet supplied to participants (translated from Swedish to English by the first author; original Swedish version available upon request) [file 13063_2021_5713_MOESM3_ESM.pdf]

LUNDS UNIVERSITET
Medicinska fakulteten

SAVE THIS INFORMATION
Train the Brain
2018-10-26

Information on participation in the "Train the Brain" study

Aim of the "Train the Brain" study
We are investigating whether physical therapy with integrated imagery training is more effective than solely physical training.

Background
Anterior cruciate ligament (ACL) injury results in instability in the knee, worse knee function, and lower quality of life. Training with a physical therapist is included in treatment, and sometimes an operation is required in which a new ligament is constructed in the knee. Rehabilitation training leads to better knee muscle function and stability, which makes it easier to complete tasks done in daily life or during sport and/or exercise. Despite training, muscle function does not always return to the same level in the injured leg as in the uninjured leg. More effective training programs are needed to further improve muscle function.

Invitation to participate
Patients that are currently undergoing rehabilitation with a physical therapist for an ACL injury (with or without operation) and that fulfill certain requirements are extended an invitation to participate.

How does the study work?
Training: For 12 weeks you will be treated either with physical therapy with integrated imagery, or with only physical therapy training with your physical therapist. This assignment will be done randomly. During the 12-week period, you should do your rehabilitation training at the clinic with your physical therapist at least 6 times total and train at least 2 times per week at home. A sport psychologist specializing in imagery will attend at least 3 trainings with your physical therapist if you are included in the physical training plus imagery group.
Assessment: We will measure your muscle strength and you will have the opportunity to do movements similar to those in your daily life, as well as in a sport/exercise context. Some tests will be filmed so that we can later assess the movements. Muscle function assessment takes approximately 1 hour. You will receive questionnaires via email regarding how you perceive your knee. These should be answered electronically and take approximately 20 minutes to complete. Assessment occurs twice, once when you are included in the study, and once again after 12 weeks of training. You will again respond to a questionnaire when it has been one year since your knee was injured or operated on. If required, we may wish to access your medical charts in regards to information related to your knee injury, for example which structures in the knee are injured besides the ACL (meniscus or other ligaments), and whether or not you had an operation.

What are the risks?
The physical training includes no more risk that normal clinical treatment. The assessments include no risks, as the movements include those that are done in daily activity or in your rehabilitation activities. Testing muscle strength can include soreness, but is not dangerous.

Are there benefits?
You will receive a thorough assessment of your muscle function several times during your rehabilitation. Those that are assigned to receive only physical training will be offered the opportunity to learn imagery training following completion of the 12 month follow-up.

Handling of data and confidentiality
Your answers and results will be treated in such a way as no unauthorized persons can access them. Every participant is assigned a code number. Data is then registered on a testing protocol sheet and in a computer. In databases, only the code number will be included. It will not be possible to identify results from a specific individual in the summary of results. Names and personal identification numbers will only be available on a separate lit for the authorized researchers. Participants' execution of certain tests will be filmed in order to assess movement quality. Each film is coded with the participants' code number so that no identification can be done, and will be stored locked separately from the code key. Data is only available to the authorized researchers. In this study, personal information will be registered. Lund University is responsible for your personal data. According to the EU's General Data Protection Regulation (GDPR), you have the right to access your personal data at no cost, and, if necessary, correct inaccuracies. You can also request that personal data be deleted and that handling your personal data be limited. If you would like to access your personal data, contact Eva Ageberg (eva.ageberg@med.lu.se, 0702-93 85 22). The data protection agency can be contacted at dataskyddsombud@lu.se or by telephone: 046-222 00 00. If you are dissatisfied with how your personal information is handled, you have the right to register a complaint with the Swedish Data Protection Authority, which is the regulatory authority. Information regarding handling of personal data is available on Lund University's website: http://www.forskningsetik.lu.se/forskningsetisk-information/personuppgiftsbehandling.

How do I get information about the results of the study?
Results will be summarized and sent to a scientific journal for publication. Contact the authors if you are interested in results of the study.

Insurance and compensation
Compensation for this study will not be offered. You are covered by the Swedish patient insurance in case of unforeseen events during the assessments.

Voluntary
Participation in this project is voluntary and you have the right to stop your participation at any time and without giving a reason. Should you decide to not participate or to end your participation, this will not influence your treatment. If you agree to participate in the study no, but choose to end your participation later, please do so by contacting one of the researchers below.

Responsible researchers
If you have further questions, please contact the responsible researchers (see contact information below).

Eva Ageberg, professor
Primary investigator
Department of Health Sciences
Lund University
E-mail: eva.ageberg@med.lu.se
Telephone: 046-2224943

Simon Granér, PhD, Senior Lecturer
Department of Psychology
Lund University
E-mail: Simon.Graner@psy.lu.se
Telephone: 046-2228776

Niklas Cederström, Doctoral candidate
Department of Health Sciences
Lund University
E-mail: niklas.cederstrom@med.lu.se
Telephone: 0725-602515

LUNDS UNIVERSITET
Medicinska fakulteten

TWO COPIES PROVIDED.
PARTICIPANT SAVE ONE COPY.

Train the Brain
2018-10-26

Consent Form

I have received information regarding the "Train the Brain" study.
I have also received information indicating that participation is voluntary and that I may end my participation at any time without giving a reason.
I agree to allow access my medical journal if necessary to get information about my knee injury.

____
Signature of participant

____
Name of participant (printed), place, date

____
Participant's personal identification number

____
Telephone number (mobile)

____
E-mail address

____
Signature of information provider

____
Name of information provider (printed), place, date
