# Supplementary material for: Motor Imagery to Facilitate Sensorimotor Re-Learning (MOTIFS) after traumatic knee injury: study protocol for an adaptive randomized controlled trial
Source: Trials. 2021 Oct 21;22:729. doi: 10.1186/s13063-021-05713-8 (PMC8532360; doi:10.1186/s13063-021-05713-8)
Supplement: Supplementary file 4 — Additional File 4: Appendix 1 - Detailed description of Care-as-Usual and PETTLEP training, and practical use of the MOTIFS model [file 13063_2021_5713_MOESM4_ESM.pdf]

Appendix for Motor Imagery to Facilitate Sensorimotor Re-Learning (MOTIFS) After Traumatic Knee Injury: Study Protocol for an Adaptive Randomized Controlled Trial

Detailed description of Care-as-Usual and PETTLEP training, and practical use of the MOTIFS model

Niklas Cederström[a], Simon Granér[b], Gustav Nilsson[c], Rickard Dahan[d], Eva Ageberg[a]

[a] Department of Health Sciences, Lund University, Margaretavägen 1B, 222 40, Lund, Sweden
[b] Department of Psychology, Lund University, Allhelgona Kyrkogata 16a, 223 62, Lund, Sweden
[c] Malmö Idrottsklinik, Kalendegatan 20, 211 35, Malmö, Sweden
[d] Kulan Idrottsskadecentrum, Eric Perssons väg 5, 217 62, Malmö, Sweden

This appendix includes further description on the following topics:

- Section 1.1 - Practical instructions in how to use the MOTIFS model, with instructions, as well as a theoretical practical example
- Section 1.2 - Detailed description of the MOTIFS model, including further practical descriptions
- Section 1.3 - Detailed description of the theoretical basis of Care-as-Usual training, as used in the design of the MOTIFS model
- Section 1.4 – Detailed description of the PETTLEP model of motor imagery, as used in the design of the MOTIFS model
- Section 1.5 – Description of the novelty of the MOTIFS model in contrast to commonly used rehabilitation practices

1.1 Practical description of how to work with the MOTIFS model for practitioners

Working with the Motor Imagery to Facilitate Sensorimotor Re-learning (MOTIFS) model builds upon existing rehabilitation strategies with integrated DMI. The principles for using the MOTIFS model in a clinical environment are applied in a series of steps which aim to aid in increasing individualized realism and meaning for a patient executing rehabilitation movements (Figure 1).

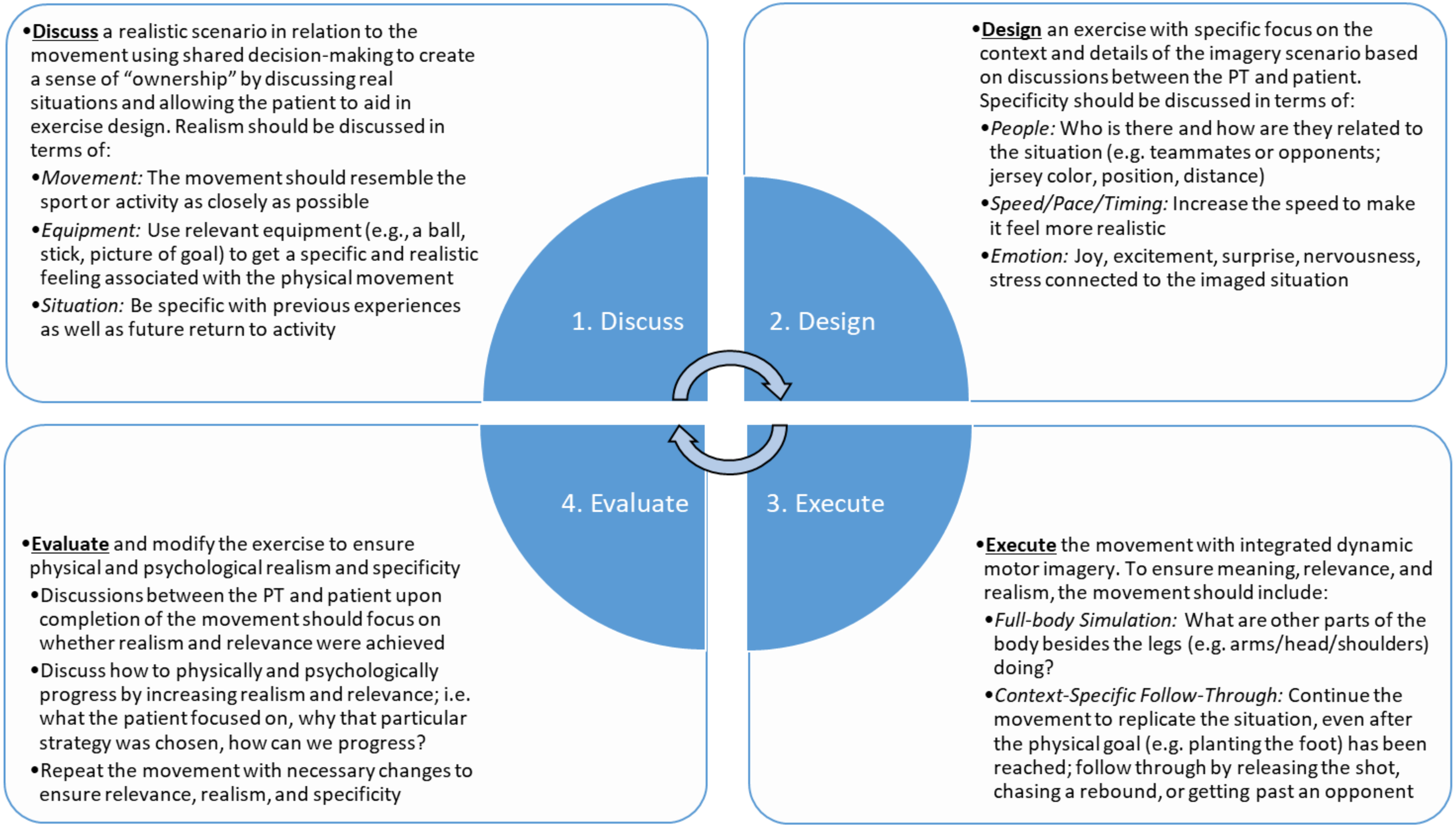

Figure 1 - Flow chart for clinical use of the MOTIFS model, and details to aid in achieving an individually relevant and context-specific exercise

1. Discuss: The treating PT begins by discussing the rehabilitation movement with the patient, including instructions in how to correctly execute the movement. Instructions are given at the discretion of the PT to ensure proper execution and quality. This may be done by applying principles of the gait cycles support (single-leg standing and single-leg squat) and toe-off phases, and a dynamic combination in a complex movement (Table 1 – "Care-as-Usual Training" column). It is important to distinguish between describing a "movement" and instructing an "exercise." In the MOTIFS model, movements are the focus in this beginning stage, as the patient will be involved in designing the exercise aiming to train a particular movement. Following instructions, the PT and patient discuss how the movement relates to the patient's sport or physical activity, with particular focus on allowing the patient to decide. Practically for the PT, this may require some effort to not give explicit examples. The patient may not immediately have an answer, though the PT has an appropriate example. The exercise given for a toe-off movement in Table 1 may provide an example: a soccer player may not immediately associate a toe-raise with a header, but the PT knows that it is appropriate. The course of action is not to immediately give the answer, but to show the movement, allow the patient to attempt the movement and add a ball to provide some context (e.g. "if the ball comes towards you at this height, show me how you would get up to that ball"). By discussing specific details of a situation and using relevant equipment, realistic aspects of the imagery situation can be increased to create a more meaningful simulation designed in part by the patient.
2. Design: Once a sport-specific situation has been identified, and at the discretion of the PT depending on physical limitations and safety, the imagery complexity can be modified. This is done using the principles outlined in section 1.2 and in the protocol for this RCT ("Table 2 – Principles utilized in the MOTIFS model"). The imagery situation is designed by discussing with the patient different technical and tactical aspects, specific relevant individuals (e.g. a teammate or opponent), speed and timing, and the emotions connected with the situation. It is important to ensure a dialogue with the patient by asking what they are thinking about, describe their feelings, where they are aiming, and the strategies and reasoning for reacting to the imagery situation in a particular way.
3. Execute: The movement is then executed, integrating dynamic motor imagery into the rehabilitation movement to create a situation which both physically and mentally simulates a sport- or activity-specific situation. Focus should include creating meaning and relevance by utilizing the whole body to simulate the movement. It is also important to include a follow-through, thinking of the next step of the movement in that particular situation, as opposed to stopping the simulation once the physical goals have been met. Encouraging follow-through can include asking them to pass or shoot the ball against a wall following completion of the targeted movement. For example, a soccer player may kick the ball to a point on the wall following completion of a squat movement, with relevant questions being asked such as "what do you do with your arms? Are there other players around you? What about your other leg? Where are you aiming? Who is standing in goal?"
4. Evaluate: Upon completing the movement, the PT should discuss with the patient whether it was a realistic and relevant movement "for your style of play," including questions about how to increase these aspects. The goal of MOTIFS training is to progress both physical and psychological complexity in order to prepare the injured person for return to activity. The evaluation should therefore include discussions of progression towards more demanding activity-specific exercise situations. In order to reflect this, the process is repeated with the agreed upon modifications. Modifications may include modulating the complexity of the imagery to fit the abilities of the patient and the rehabilitation goals on which the PT wishes to focus (a less complex image can be used to ensure proper movement quality and safety; a more complex image can be used to challenge the patient). By including more aspects of DMI, it is possible to create a more functionally equivalent movement that will likely include higher physical and psychological demands. It is important here that the PT does not allow for a reduction in movement quality in regards to the targeted rehabilitation movement while allowing for arm or torso movement in the sport-specific situation. If movement quality is not maintained, complexity of the imagery may be reduced. The PT may also reduce the physical demands and increase the psychological aspect in order to train reaction, for example.

Practical Example: As a practical example, consider the soccer player in Table 1 ("MOTIFS" column). Assuming that he is in the earlier stages of the rehabilitation process and has only just begun directional change movement, he may execute a complex movement in which he is simply planting and pushing off in another direction. He should be shown the movement and instructed in proper execution. Following this, a discussion with the PT reveals that he is a defensive player and believes that a relevant movement will be receiving a ball and dribbling past an opponent. He first attempts the movement with few to no DMI aspects, with only the ball placed on the ground in front of him, after which the realism and relevance is discussed. He may decide that it is more relevant to receive the ball while taking a backwards step before moving forwards. This provides a context- and activity-relevant modification. In the next set, imagery aspects are added, by passing the ball after "cutting" around a cone, thinking specifically about who is receiving the pass and where potential opposing players are. Throughout the course of rehabilitation, this can be made more complex by increasing speed, cutting past the PT instead of a cone, eventually adding light contact (i.e. he pushes away the PT), and increasing stress by setting a time limit, a randomized passing pattern (i.e. PT yells left, right, center), varying the speed and angle of the received pass (i.e. left, right, in the air, interception), or the PT may act as an opposing player and try to take the ball. At the center of the rehabilitation exercises with integrated DMI are the MOTIFS principles of achieving and maintaining realism and individualized specificity using full-body simulations with context-specific follow-throughs.

1.2 The MOTIFS Model

The MOTIFS model includes all aspects of CaU rehabilitation while simultaneously integrating psychological aspects into them (Table 1 "MOTIFS" column). This allows for sport-specificity not only in terms of the movement, but also in terms of performance- and meaning-based realism based on their own previous experiences. The injury and all of the physical aspects of rehabilitation are taken into account in order to ensure that the PT goals are fulfilled. The MOTIFS model also includes more situation-specific and individual performance goals, such as the ability to perform a specific task (i.e. shooting/blocking). The execution of the MOTIFS exercises are based on those that the treating PT would typically prescribe for that particular injury and patient. The difference is in terms of the focus and context in which the exercises are performed. MOTIFS focuses on creating understanding of the movement not only in terms of the physical and rehabilitative movement, but also the technical/tactical aspects, as well as the context-specific execution for the situation which the individual deems relevant for that movement. This also includes a follow-through movement, for example if the prescribed movement aims to improve a cutting movement, a soccer player may see it as a shooting situation, continue the movement by taking an extra step around the 'opponent' and follow through on the shot. The particular situation is dependent on the patient and what he or she decides is relevant, which constitutes another difference: that the patient is involved in the design of the exercise itself. In the MOTIFS model, the PT explains the movement broadly and then discusses with the patient how that movement is relevant, allowing the patient to decide how to incorporate their sport into the movement, which provides a sense of 'ownership' of the exercise. This is in contrast to typical rehabilitation programs in which the PT prescribes an exercise to fulfill their goals, without consulting the patient in how to make it individually relevant.

1.3 Care-as-Usual rehabilitation following traumatic knee injury

The loading phases described in Table 1 ("Care-as-Usual Training" column) are examples of basal positions and neuromuscular principles which the physical therapist (PT) may use to prescribe exercises, the intensity and speed of which are modified at the PTs discretion. Care-as-Usual (CaU) rehabilitation tends to focus on physical execution of tasks from a movement-based perspective based on the injury being treated. This includes aspects of postural control, balance and strength in multiple joints/muscle groups and functional positions. The goal is to gain the ability and understanding of the rehabilitation movement in order to rehabilitate the injured joint, thereby fulfilling an outcome-oriented goal (often return to sport or activity). Patients tend to be involved in the decision-making process to the extent that they can give perspectives on broad treatment options, though specifics are often the responsibility of the PT.

Table 1 - Examples of commonly used rehabilitation training principles and focuses in Care-as-Usual training, and examples of modifications to integrate DMI using the MOTIFS model

	**Care-as-Usual Training**		**MOTIFS**	
	Neuromuscular training aiming to improve sensorimotor control and functional stability; exercises involve multiple joints and muscle groups in weight-bearing positions, with focus on correct movement quality, including postural orientation		*Participants complete Care-as-Usual exercise, integrating DMI with focus on sport-specific situations, using equipment to increase realism. Throughout the movement, the PT ensures proper movement quality*	
**Single-Leg Standing**	Single-leg standing (corresponding to late mid-stance) in which function and postural control is challenged by the movement of other parts of the body. Main focuses:<br>• Even weight distribution over the whole foot (all toes, the heel, and the floor), avoiding excessive pronation or dropping of the medial arch of the foot<br>• Knee joint in line with the hip and foot (activate the quadriceps and hamstrings, as well as gluteal muscles), that is maintaining postural orientation of the hip, knee, and trunk, such as avoiding knee medial to foot position, and/or deviation of the pelvis and/or trunk in any plane<br>• Neutral low back relative to pelvis and chest (activate core and lateral gluteal muscles)	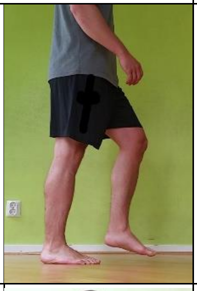	The participant focuses on maintaining balance while receiving a volley, looking for where to pass next and from where opponents might be coming	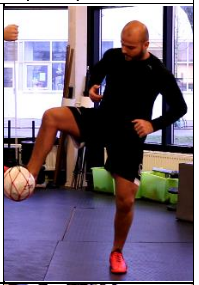
**Single-Leg Squat**	Single-leg squat (corresponding to early mid-stance) in which function and postural control is challenged while training the ability to absorb and create power. Main focuses:<br>• Even weight distribution over the whole foot (all toes, the heel, and the floor), avoiding excessive pronation or dropping of the medial arch of the foot<br>• Knee joint in line with the hip and foot (activate the quadriceps and hamstrings, as well as gluteal muscles), that is maintaining postural orientation of the hip, knee, and trunk, such as avoiding knee medial to foot position, and/or deviation of the pelvis and/or trunk in any plane<br>• Neutral low back relative to pelvis and chest (activate core and lateral gluteal muscles)	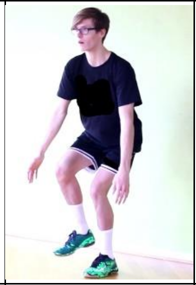	The participant focuses on the approach, shot, and follow-through in a free kick situation, thinking about following the ball after release	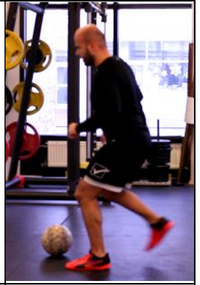
**Toe-Off**	Single-leg toe-off in which function and postural control is challenged in a triple extension movement (plantar flexion, knee extension, hip extension). Main focuses:<br>• Even weight distribution over forefoot (ensure contact with the big toe and second toe), avoiding a lateral push-off<br>• Active and simultaneous extension of the hip, knee and ankle joints (knee joint in line with the hip and foot; knee extension to approx. 0°)<br>Neutral pelvic control and neutral low back (activate core and gluteal muscles, avoiding pelvic and/or trunk deviations in any plane)	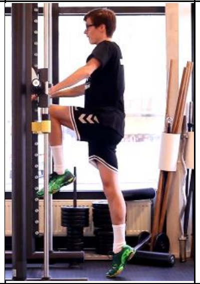	The participant focuses on reaching up towards the ball, thinking of where to head the ball, what the arms are doing, and scoring on the opposing goalie	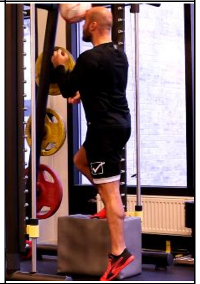
**Complex Movement**	Complex movements are based on and combine the loading-phase movements (toe-off, single-leg standing, single-leg squat) which are performed with simultaneous directional changes at progressively higher speeds. The aim is to control movement quality during acceleration and deceleration. It is first trained in a single plane (sagittal or frontal), as well as vertically (jumping), which later intensifies to directional changes in different and/or multiple planes, as well as allowing pivoting movements.	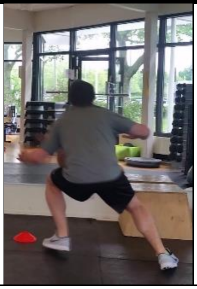	The participant focuses on moving around an opponent, in order to intercept or receive the ball, as well as thinking of whether to dribble forward, or look for a passing opportunity	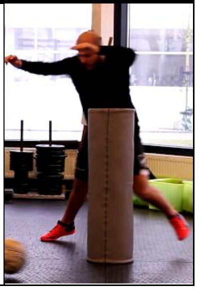

1.4 The PETTLEP model of motor imagery

PETTLEP is a model of dynamic motor imagery (DMI) developed to create functionally equivalent movements, thereby allowing more thorough recall of sport-specific and realistic situations. The model is built on the 7 aspects which make up the acronym PETTLEP: Physical, Environmental, Task, Timing, Learning, Emotion, and Perspective (Table 2). Each aspect aids in increasing functional equivalence by discussing with the person each of these aspects.

Table 2 – Description and examples of PETTLEP motor imagery used as a performance enhancement tool in uninjured athletes

**P**hysical	The physical movement to be imaged should be included in the DMI image, and be as similar as possible to actual execution. One can use additional movements, such as arm and head movements in a shooting situation, to increase physical similarities to actual physical activity execution.<br>*Example: Psychologically imaging a goal and goalie, and physically taking steps forward and executing an actual shot (against a wall, for example)*
**E**nvironmental	The physical activity environment should be replicated and/or imaged as much as possible. This may include imaging on the grass if possible (i.e. replication), or by recalling an image of the noise your teammates make on the bench (i.e. imagery).<br>*Example: The smell of the grass, wearing the same training clothing, using equipment, imaging the training center, how much time is left on the clock*
**T**ask	Imagery should be relevant to the physical activity being performed. A soccer player should image a situation which is relevant to his or her specific position (e.g. goalie vs striker) and style of play. One creates meaning in this way by imaging individualized and task-specific situations.<br>*Example: For a soccer player, a header situation may be relevant, but it may be contextually different for an offensive player (shot on goal) and a defensive player (bring down a ball to the feet)*
**T**iming	The imager should be aware of the timing in a movement. A soccer player should think about how long an approach takes and how many steps in a free kick situation. This timing can be used as a reference to create the speed and timing in the imagery exercise.<br>*Example: A free kick approach takes 2 seconds with 3 steps, so the imagery should include 3 steps over a 2-second period*
**L**earning	The imager should have the perspective that this is a possibility to learn or improve a skill in their sport, as well as physical movements. Corrections following a mistake are encouraged as a positive aspect of the development process.<br>*Example: Identify that the shot was too low and correct on the next repetition*
**E**motion	Happiness, excitement, frustration, and nervousness are a natural part of sport, and should be included in the imagery exercise.<br>*Example: Increase stress using time limitations (10 repetitions in 30 seconds), targets (a series of dots on the wall or a picture of a goal), or increased speed and pulse; image feelings of relief after scoring a goal in the final minutes*
**P**erspective	The patient should image an activity-specific and relevant situation as though they are performing the movement themselves (that is, first-person perspective). Focus should be on the physical (kinetic and tactile)<br>*Example: How does it feel in my legs when I shoot? Where am I looking?*

When using the PETTLEP model, the degree of complexity depends on the goals and/or abilities of the imager, and can be increased or decreased by modifying the number of PETTLEP aspects included in the imagery exercise. Constant evaluation is necessary to make the situation as realistic as possible. This is done by asking questions about the situation (potentially regarding who the goalie was, where are your teammates, which stadium are you playing in, did you celebrate after the goal?), as well as by encouraging relevant physical movement (what are your arms doing during the shot; where are you looking; did you follow-through with your foot and eyes after the pass?).

An important challenge for the practitioner is to encourage self-regulation. This includes allowing the imager to decide him- or herself what the situation is (that is, not trying to create an

exercise for the imager, but allow them to decide what is relevant). After a completed set, the imager should be asked if it felt realistic, if that is something they would actually do in a competition or training situation; if not, discuss how to modify it to increase individual realism and relevance.

1.5 Novelty of the MOTIFS Model

The MOTIFS model is based on the PETTLEP model of DMI. There are some key differences to take note of, however, which distinguish it from the MOTIFS model. PETTLEP is typically used in an uninjured population and therefore is not adapted specifically to rehabilitation exercises and environments. The MOTIFS model is based specifically on including aspects of PETTLEP DMI into existing exercises with the main aim of rehabilitation following a traumatic injury. This means that MOTIFS includes aspects of rehabilitation such as balance and strength in functional positions, which are not necessarily a focus of PETTLEP imagery.

A potential point of contention may be the focus of attention in CaU compared to MOTIFS training. While it may be true that current rehabilitation programs do not use a strictly internal focus of attention (that is, complete focus on activating specific muscles and/or position of joints in relation to one another), it is arguable that MOTIFS has a focus of attention which is more external. Rehabilitation exercises are often described by instructing the injured person to reach their knee towards the wall, or stretch towards the ceiling. This is indeed an external focus of attention, though it still has as its main focus on body positioning and muscular activation. In the MOTIFS model, on the other hand, the focus is external in the sense that the focus is on successful task completion. Stretching towards the ceiling may be replaced by stretching your body up towards the ball and feeling the headed ball go into the goal. The physical execution may look similar, but the focus is quite different.

The MOTIFS model is based on both CaU rehabilitation programs and the PETTLEP model of motor imagery, modifying each in a novel training model focusing on realism and specificity to create context-specific simulations of relevant sport or physical activity situations (Table 3).

Table 3 – Clarification of basis and focus of Care-as-Usual, PETTLEP motor imagery, and MOTIFS training models

Basis	Focus			CaU	PETTLEP	MOTIFS
Physical Therapy-Based	Injury Rehabilitation			X		X
Physical Therapy-Based	Postural Control			X		X
Physical Therapy-Based	Balance			X		X
Physical Therapy-Based	Strength			X		X
Physical Therapy-Based	Multiple Joints and Muscle Groups			X		X
Physical Therapy-Based	Functional Positions			X		X
Psychologically-Based	Individualization	Injury-based		X		X
Psychologically-Based	Individualization	Experience-Based			X	X
Psychologically-Based	Individualization	Goal-Based	Outcome	X	X	X
Psychologically-Based	Individualization	Goal-Based	Situation-Specific		X	X
Psychologically-Based	Physical Simulation (Task-specific Movement)				X	X
Psychologically-Based	Environmental (People/Place/Equipment)				X	X
Psychologically-Based	Task (Sport-specific Realism)				X	X
Psychologically-Based	Timing				X	X
Psychologically-Based	Learning	Technical/Tactical Movement		§	X	X
Psychologically-Based	Learning	Context-Specific Understanding		§	X	X
Psychologically-Based	Learning	Physical Movement Understanding		X	X	X
Psychologically-Based	Learning	Rehabilitative Movement		X		X
Psychologically-Based	Emotion				X	X
Psychologically-Based	Perspective				X	X
Psychologically-Based	Shared Decision-Making	Broad Treatment Options		X		X
Psychologically-Based	Shared Decision-Making	Exercise-Specific Design				X
Psychologically-Based	"Ownership" of Rehab					X
Psychologically-Based	Context-Specific Follow-Through of Rehab Exercise					X
Psychologically-Based	Motivation	Intrinsic (Inherent Satisfaction)			X	X
Psychologically-Based	Motivation	Extrinsic (Outcome-Oriented)		X	X	
Psychologically-Based	Focus of Attention*	External (Task-Based Execution)			X	X
Psychologically-Based	Focus of Attention*	Internal (Own Body Positioning)		X		
Activity - Specific	Movement			X	X	X
Activity - Specific	Performance				X	X
Activity - Specific	Meaning				X	X
Activity - Specific	Equipment			§	X	X

§ These aspects may also be present in CaU rehabilitation training, though the focus tends to be on physical preparation, and not particularly on psychological learning

** MOTIFS and PETTLEP focus is arguably more external than CaU given that the rehabilitation-specificity in CaU is typically still in reference to body-positioning, whereas MOTIFS and PETTLEP focus on environmental and situation-specific factors to a greater degree*
